# Resting-State EEG Power Spectral Density Analysis Between Healthy and Cognitively Impaired Subjects

**DOI:** 10.3390/brainsci15020173

**Published:** 2025-02-10

**Authors:** Katherine F. Walters, Rohit Shukla, Vivek Kumar, Shannon Schueren, Hariom Yadav, Nathan D. Schilaty, Shalini Jain

**Affiliations:** 1 NeuBaC Laboratory, Department of Neurosurgery and Brain Repair, Center for Neuromusculoskeletal Research, University of South Florida, Tampa, FL 33620, USA; 2 USF Center for Microbiome Research, Microbiomes Institute, Department of Neurosurgery and Brain Repair, University of South Florida Morsani College of Medicine, Tampa, FL 33620, USA; 3 NeuBaC Laboratory, Department of Medical Engineering, University of South Florida, Tampa, FL 33620, USA

**Keywords:** mild cognitive impairment (MCI), electroencephalography (EEG), cognitive decline, power spectral density (PSD), neurodegenerative diseases

## Abstract

**Background/Objectives**: This study evaluates the potential of electroencephalography (EEG) as a noninvasive tool for distinguishing between healthy individuals (*n* = 79), those with mild cognitive impairment (MCI; *n* = 36), and dementia patients (*n* = 7). **Methods**: Using a 14-channel Emotiv EPOC-X headset, we analyzed power spectral density during a 2-min eyes-closed resting state. **Results**: Our results demonstrated that while EEG effectively differentiated dementia patients from healthy controls, it did not show significant differences between MCI and healthy controls. This indicates that EEG holds promise for identifying advanced cognitive decline but faces challenges in early-stage detection. **Conclusions**: The study contributes to the growing body of literature by highlighting EEG’s potential as a cost-effective alternative to invasive diagnostic methods while also identifying the need for larger sample sizes and task-oriented approaches to improve its diagnostic precision.

## 1. Introduction

Older adults face a significant burden of age-related cognitive decline, with aging recognized as the primary risk factor for most neurodegenerative diseases, including Alzheimer’s disease (AD). However, other factors also contribute to the development and progression of AD. Genetic predispositions, such as carrying the APOE-ε4 allele, are well-documented risk factors. Lifestyle behaviors, including physical inactivity, smoking, poor dietary habits, and limited cognitive engagement, further increase susceptibility to the disease. Additionally, environmental exposures, such as air pollution and heavy metal toxins, have been linked to neurodegenerative processes. These factors may act independently or interact with aging and one another to influence the likelihood and severity of AD [1]. AD, which is progressive, untreatable, and irreversible, is the most common form of dementia among older adults, accounting for 60–80% of cases. It is characterized by the buildup of abnormal proteins, such as amyloid-beta (Aβ) and phosphorylated tau (ptau), in the brain, leading to the degeneration of nerve cells. Worldwide, more than 55 million people suffer from AD, including 6.9 million Americans aged 65 and older [2]. Alarmingly, projections suggest that the number of cases could rise to 13.8 million by 2060 if no medical interventions are made [2,3]. The absence of effective treatments places a considerable burden on patients, their families, society, and healthcare systems.

Currently, Alzheimer’s disease is typically diagnosed at advanced stages when it is irreversible, and available treatments are limited to managing symptoms [4,5,6,7]. However, the neuropathological changes associated with AD begin years before the onset of noticeable symptoms [8]. These early changes often manifest in a preclinical stage called mild cognitive impairment (MCI), which can eventually progress to dementia [9,10]. MCI serves as a critical precursor to AD, with an annual progression rate estimated at 10–15% [11]. Identifying AD during the MCI stage is essential for slowing cognitive decline and delaying dementia onset. Despite this, current diagnostic methods are invasive, expensive, and not easily scalable. Techniques such as positron emission tomography (PET) scans, cerebrospinal fluid (CSF) analysis, blood tests, and magnetic resonance imaging (MRI) are frequently used [12]. While these approaches provide valuable information, their limitations—high costs, invasiveness, and limited accessibility—underscore the urgent need for alternative diagnostic tools [12,13,14].

Electroencephalography (EEG) has emerged as a promising noninvasive and portable method for monitoring neural activity through cortical brain recordings. Recent studies indicate that EEG can effectively distinguish between healthy individuals, those with MCI, and individuals with dementia [15,16,17,18,19]. Specific EEG patterns—such as increased delta and theta oscillations coupled with decreased alpha and beta rhythms—have been associated with Alzheimer’s disease and MCI [15,20,21,22,23]. Furthermore, reduced complexity and coherence in EEG recordings, as well as certain frequency ratios (e.g., theta-to-gamma wave ratios), have shown potential as biomarkers for AD [22,24,25,26,27,28,29,30]. These findings highlight EEG’s ability to capture neural changes indicative of early cognitive decline.

Despite the promising nature of EEG-based biomarkers, challenges remain in translating research findings into clinical practice. Many studies are limited by small sample sizes, often involving fewer than 100 participants, which restricts their generalizability and limits the statistical power to identify robust biomarkers [22]. Larger-scale studies, involving several hundred to thousands of participants across diverse populations and clinical settings, are needed to validate these findings and ensure their applicability in real-world scenarios. While specific EEG metrics such as power spectrum and entropy have been widely studied [31,32], their utility in diagnosing MCI and AD requires further validation. Addressing these gaps, this research aims to evaluate EEG characteristics as potential biomarkers for differentiating cognitively impaired individuals, particularly in early-stage MCI detection, from healthy controls. The analysis will focus on changes in frequency or power spectrum—either individually or in combination—captured during a 2-min resting-state EEG with eyes closed. By leveraging these EEG biomarkers, this study seeks to enhance early Alzheimer’s disease detection and support the development of more effective interventions. We hypothesize that the average power spectral density (PSD) of cortical waveforms will show distinct variations across cognitive states in healthy individuals, those with MCI, and participants with dementia.

## 2. Materials and Methods

### 2.1. Participant Recruitment

The data and samples for this study were obtained from the Microbiome in Aging Gut and Brain (MiaGB) Consortium as part of a pilot project. Participants were recruited from the ongoing MiaGB study, which is funded by the Florida Department of Health. This study and its EEG protocol received approval from the University of South Florida Institutional Review Board (STUDY002365), and all participants provided informed consent.

Inclusion criteria required participants to be able to speak and read English and to have either normal cognitive function, mild cognitive impairment, or dementia. Exclusion criteria included the following: (1) history of brain- or gut-related surgeries in the past five years; (2) history of cancer diagnosis or treatment (except melanoma skin cancer) in the past five years; (3) neurological disorders such as epilepsy, Parkinson’s disease, or amyotrophic lateral sclerosis; (4) antibiotic use in the past four weeks; (5) recent diarrhea, vomiting, or food poisoning within the past four weeks; (6) a history of inflammatory bowel disease; (7) a weight loss of more than 10 pounds in the last two weeks; and (8) a body mass index (BMI) greater than 45 or less than 18 kg/m^2^.

### 2.2. Cognitive Assessments

Cognitive function was assessed using the Montreal Cognitive Assessment (MoCA) [33] and other cognitive tests, including the Mini-Cog© (0–5 points; with 0–2 indicating a higher likelihood of significant cognitive impairment and 3–5 indicating a lower likelihood) [34] and the Memory Impairment Screen (MIS; 0–8 points, with 0–4 indicating possible cognitive impairment and 5–8 indicating no significant impairment) [35,36,37,38,39,40]. The MoCA is scored out of 30 points and is interpreted as follows: normal cognition (26–30 points); mild cognitive impairment (18–25 points); moderate cognitive impairment (10–17 points); severe cognitive impairment (below 10 points) [41].

The other assessments are taken by participants and informants who are family or friends of the participant and have observed their cognitive changes. The Questionnaire on Cognitive Decline in the Elderly (CDQE; 26 items) or Short Form of the Informant Questionnaire on Cognitive Decline in the Elderly (Short IQCODE; 16 items) addresses the comparison of the present memory and knowledge of the participant to 10 years prior. It covers memories about events, conversations, addresses, etc. It allows the informant to rate it on a scale: *much improved*, *a bit improved*, *not much change*, *a bit worse*, or *much worse*, all receiving a number ranking of 1, 2, 3, 4, or 5, respectively. The scoring is done by dividing the number of questions (26 for the long form and 16 for the short form). The results range from 1 to 5, with a score of 3 as *no change*, 4 as *a bit worse*, and 5 as *much worse*. For the long and short versions, the cut point for screening for dementia is 3.27/3.30 and 3.31/3.38, respectively [38,39,40,42,43]. The Eight-Item Informant Interview to Differentiate Aging and Dementia (AD8) was also taken by individuals who know the participants well. This 8-item questionnaire is a brief instrument serving to help distinguish between normal aging and dementia. The score is a sum of the “YES, a change” answers given. The breakdown of the categorization is normal cognition (0–1) or impairment in cognition (>2) [44,45].

EEG data were collected from 223 participants in the MiaGB study. Participants were categorized based on their MoCA scores into three groups: healthy controls (HC; *n* = 146), mild cognitive impairment (MCI; *n* = 65), and dementia (D; *n* = 10). The healthy controls were further divided by age into three subgroups: 55 years or younger (HC1; *n* = 11), ages 56 to 64 (HC2; *n* = 40), and 65 years or older (HC3; *n* = 95).

Given that cognitive impairment is more common in older adults, the HC3 group, hereafter referred to as “HC”, was selected for analysis. Additionally, to ensure a comparable age range with the HC group, some MCI participants were excluded, resulting in a final total of 49 MCI participants for the study.

### 2.3. Electroencephalography (EEG) Recording

A 14-channel EPOC-X wireless headset was used for EEG recordings, selected for its established reliability in similar studies [46]. The EPOC-X is also easy to set up, making it well suited for clinical settings. We employed a structured testing and acquisition software platform (Emotiv Inc., San Francisco, CA, USA) to ensure consistent data collection. EEG data were recorded using the following 14 electrodes according to the 10–20 system: AF3, F3, F7, FC5, T7, P3, P7, O1, O2, P8, P4, T8, FC6, F8, F4, and AF4. The electrodes were well-saturated with saline solution before being inserted into the EPOC-X headset. The headset was positioned so that the CMS and DRL ground electrodes were securely placed on the mastoid processes behind each ear. The remaining electrodes were arranged symmetrically and according to standard procedures. Participants were seated comfortably with a laptop computer positioned on a desk approximately 65 cm in front of them. Prior to data collection, the quality of the electrodes was checked and had to meet a threshold of 76%. After ensuring signal quality, participants remained seated and had EEG recorded for 2 min with their eyes closed (EC2min).

### 2.4. Data Processing and Statistical Analysis

Post-processing and statistical analyses were conducted using EEGLAB (v2024.0; Swartz Center for Computational Neuroscience, La Jolla, CA, USA) [47]. Within EEGLAB, data were post-processed according to industry standards. The data were band-pass filtered between 1 and 60 Hz and re-referenced to average power. EEG channels with more than 30 s of flat-line signal (>25% of the task) or those exhibiting high-frequency noise exceeding four standard deviations were excluded using the clean_rawdata EEGLAB plugin v0.31. Datasets with fewer than six channels remaining after processing were discarded. Data with brief periods of severe noise (greater than 20 standard deviations) were corrected using the Artifact Subspace Reconstruction algorithm [48]. Independent Component Analysis (ICA) was performed on each event data sample using the Infomax ICA algorithm with default EEGLAB settings, and component labeling was performed using the ICLabel toolbox [49]. Components associated with Eye, Muscle, Heart, Line Noise, and Channel Noise (>60%) were manually rejected based on ICLabel’s identification of independent component sources.

Although participants engaged in various activities, only the 2-min eyes-closed resting state (EC2min) trials were selected and reported in this manuscript to align with current trends in the recent literature. The raw EEG data were converted into spectral power using EmotivPRO software v4.5.4.567 (Emotiv, San Francisco, CA, USA). Spectral power indicates frequency and enables comparison of bandwidths across groups based on amplitude. One-way ANOVA was used for spectral analysis and other measurements such as cognitive function tests, age, and gender. Post hoc *t*-tests with Bonferroni corrections were applied when appropriate. All statistical tests were conducted with a significance level set at *p* < 0.05. All PSD values reported are averaged across the 14 electrodes, providing a comprehensive measure of the spectral power across the scalp.

## 3. Results

### 3.1. Demographic Data

After evaluating the data quality, we excluded participants (*n* = 101) who either had insufficient data or whose data quality was inadequate. The final sample sizes were as follows: HC (*n* = 79), MCI (*n* = 36), and D (*n* = 7).

We included 122 participants from the MiaGB study, comprising 77 females and 45 males. The final sample sizes were as follows: HC (*n* = 79), MCI (*n* = 36), and D (*n* = 7). Table 1 summarizes the participants’ demographics and cognitive function test results. Significant differences were observed in education levels and cognitive test scores (MoCA, Mini-Cog©, MIS, CDQE, and AD8) among the three groups: HC, MCI, and D.

### 3.2. EEG Data and Recordings

**Figure 1:**
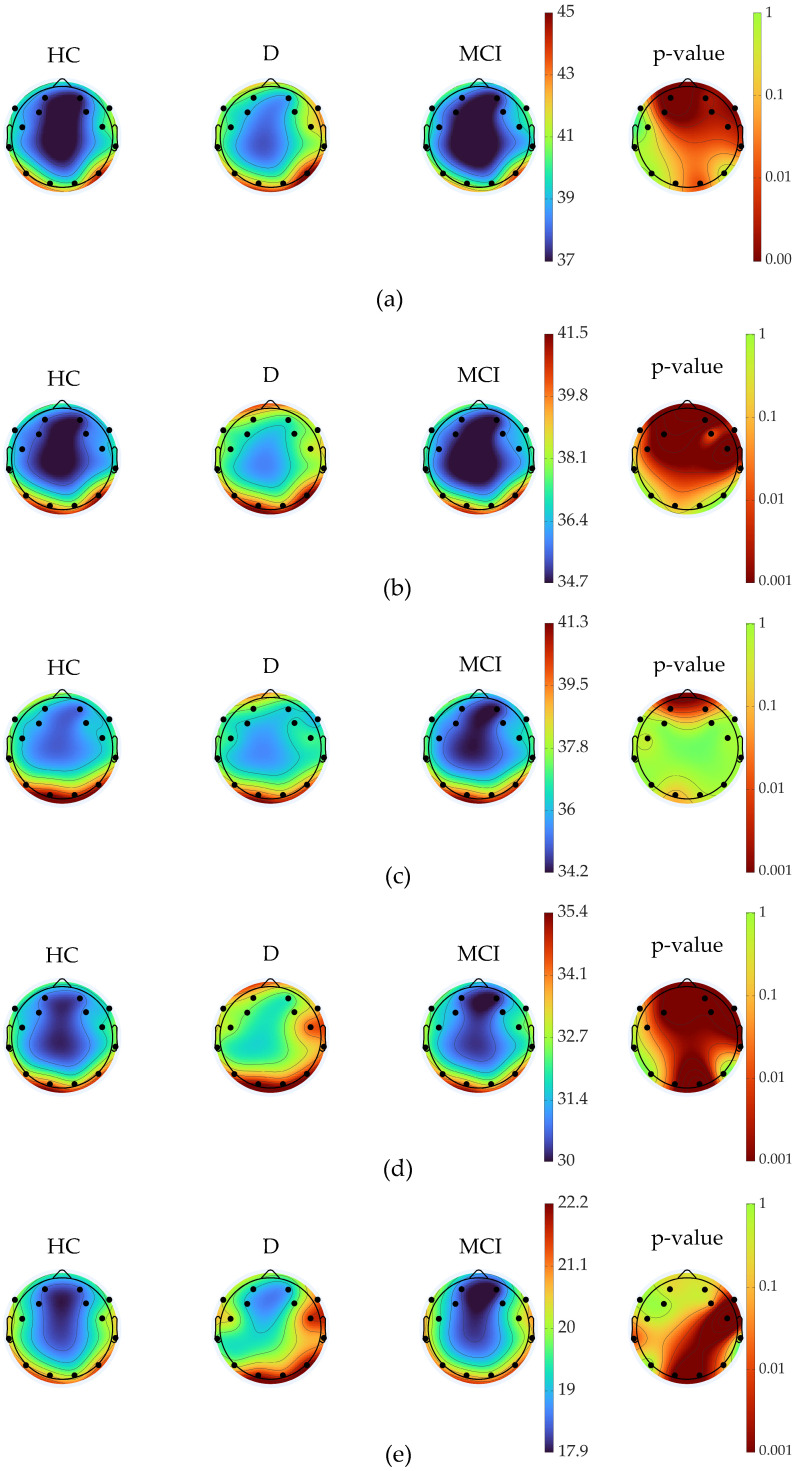
Power spectral density (PSD) differences between groups: healthy controls (HC), mild cognitive impairment (MCI), and dementia (D). PSD represents the distribution of power across different frequency bands. The images show PSD for five frequency ranges: (**a**) delta (1–4 Hz), (**b**) theta (4–8 Hz), (**c**) alpha (8–13 Hz), (**d**) beta (13–30 Hz), and (**e**) gamma (30–60 Hz). The rightmost column displays *p*-values, indicating the statistical significance of differences between the three groups (HC, MCI, and D) for each scalp region covered by the 14 electrodes. Significant differences (indicated by red/darker regions in the *p*-value maps) reflect alterations in brain oscillatory activity across the groups, with specific frequency bands showing group-specific patterns. Bonferroni correction was applied to control for multiple comparisons.

**Figure 2:**
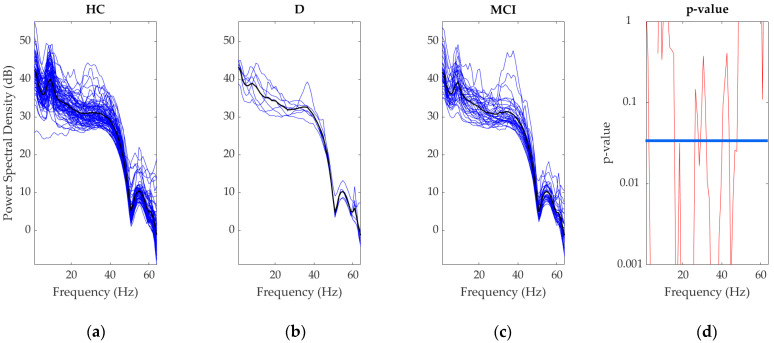
Average power spectral density for the full frequency range (1–60 Hz) across all groups (HC, MCI, D). (**a**) Healthy control average power spectral density, (**b**) dementia average power spectral density, and (**c**) mild cognitive impairment average power spectral density. In the first three plots, blue lines represent individual participant data, and the black line represents the group average for each condition (healthy controls, mild cognitive impairment, Dementia). (**d**) The *p*-values for one-way ANOVA across the full frequency range (1–60 Hz), with the blue line marking the significance threshold (*p* = 0.05). The red line indicates the *p*-value for each frequency bin, and frequencies where the red line drops below the blue threshold signify significant differences between groups. These differences suggest altered brain activity in specific frequency ranges across the three conditions. Statistical comparisons were performed using one-way ANOVA to assess differences across the groups over the entire frequency range.

EEG data were recorded across multiple frequency bands from various scalp regions. We observed significant differences in the mean values across the entire frequency spectrum among the three groups (Figure 1a; *p* < 0.05). These differences were present in all frequency bands—delta, theta, alpha, beta, and gamma (Figure 1b–e). Figure 2 shows these differences by presenting the average PSD across the frequency spectrum, along with the corresponding *p*-values.

**Figure 3:**
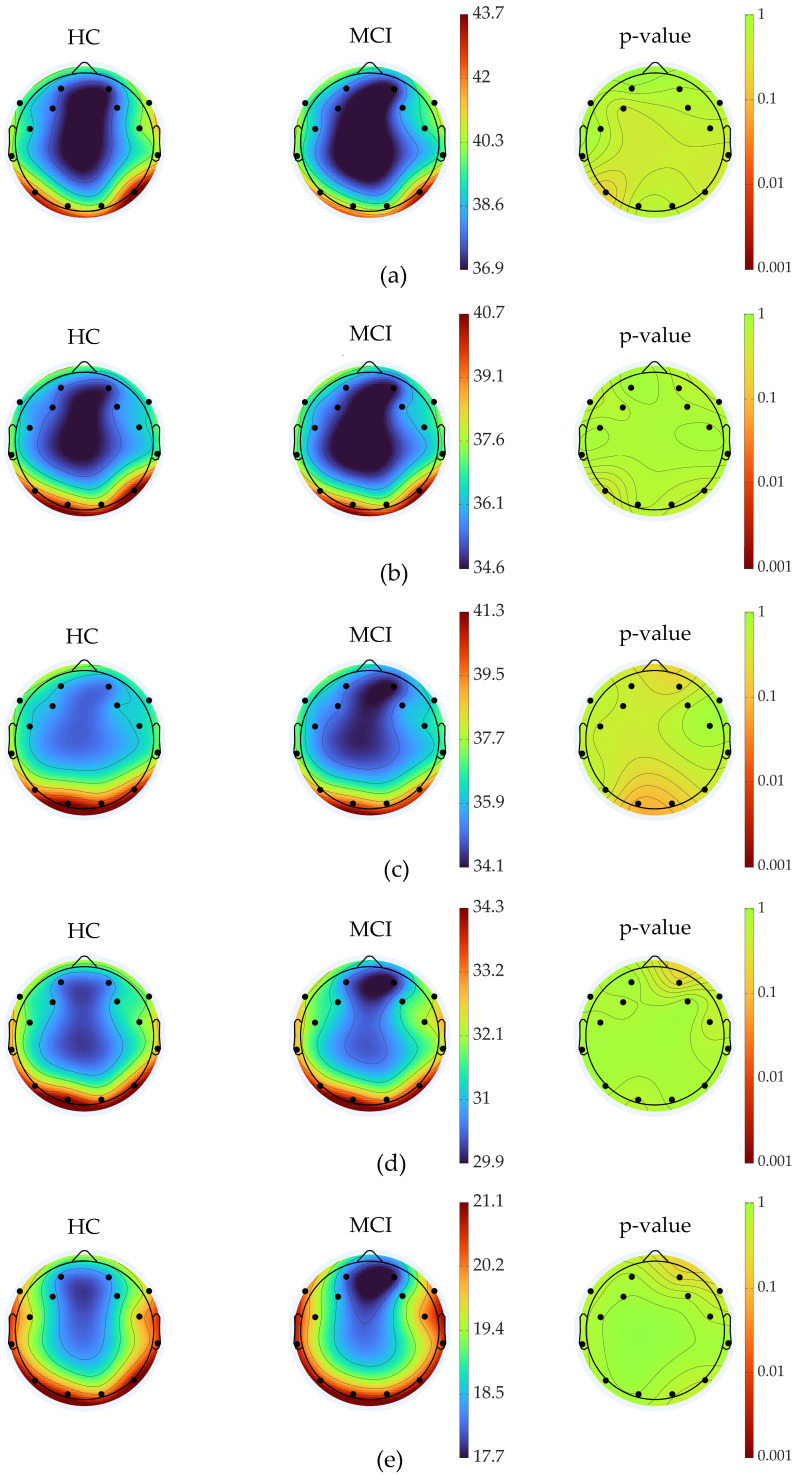
Average topography for healthy controls (HC) vs. mild cognitive impairment (MCI) with *p*-values. Topographic maps show the power spectral density (PSD) across six frequency ranges: (**a**) delta (1–4 Hz), (**b**) theta (4–8 Hz), (**c**) alpha (8–13 Hz), (**d**) beta (13–30 Hz), and (**e**) gamma (30–60 Hz). Each pair of maps compares PSD between HC and MCI groups, while the rightmost column shows the corresponding *p*-values for statistical significance across scalp regions. Areas with darker or more red regions in the *p*-value maps indicate significant differences in brain activity between HC and MCI groups.

When comparing healthy controls to participants with mild cognitive impairment, we found no significant differences in average PSD during the 2-min eyes-closed resting EC2min state (Figure 3). Additional comparisons between MCI and dementia (D) participants and between healthy controls and dementia participants also did not reveal any further differences [16,50,51].

## 4. Discussion

This study revealed that participants with dementia (D) differed from those with mild cognitive impairment (MCI) and healthy controls (HC) during a 2-min eyes-closed resting task (EC2min). However, no differences were found between the MCI and HC groups. These results partially support the hypothesis that the average PSD of cortical waveforms during a 2-min eyes-closed resting state can distinguish between different cognitive statuses. Table 1 shows that, in addition to differences in cognition tests between healthy controls and individuals with cognitive decline, there were also variations in education levels among participants. These findings suggest that education level is a major factor in cognitive decline, as participants with dementia had lower levels of education compared to the other two groups. This observation is consistent with research indicating that the length of education is related to cognitive decline [52,53]. Previous studies suggest that more years of education are associated with better cognitive performance in older adults [52,53].

The results from the D group participants in this study seem to contradict previous research. Figure 1 and Figure 2 illustrate the differences between the three groups. The observed differences are primarily attributed to the discrepancies between the dementia group and the other two groups, which show no differences among each other (discussed below). It appears that the dementia group influences these differences across all bandwidths and multiple scalp electrodes. This inconsistency might be due to several factors, including variations in the number of electrodes used, the duration of the eyes-closed task, baseline differences in resting state activity, comorbidities, or medication use.

Despite the significant changes observed when comparing HC, MCI, and D groups, participants with dementia showed higher amplitude across all frequency bands. This is contrary to previous studies, which generally report that dementia patients have lower PSD in higher frequency bands compared to healthy controls [16]. Typical differences associated with normal aging, cognitive impairment, and dementia diagnosis vary depending on the frequency bandwidth, as indicated by Krothapalli et al. (2024) [50]. Although not statistically significant, the trends between the HC and MCI groups were notable, especially in the alpha frequency range (see Figure 3d). MCI participants displayed a reduction in PSD in the alpha wave frequency in the occipital region compared to healthy controls, a pattern similar to that seen in Alzheimer’s disease patients compared to healthy controls [15,16,54,55]. Participants were instructed to close their eyes to simulate a resting state. Typically, this would not result in higher amplitudes in beta and gamma bandwidths [24]. However, the higher amplitude observed in the D group contradicts previous literature, which typically reports that dementia patients exhibit lower amplitude in these bandwidths compared to healthy controls [56,57]. The recent literature has emphasized the resting state in EEG studies to categorize stages of cognitive decline [58]. The resting state, also known as the Default Mode Network (DMN), involves bilateral and symmetrical cortical areas, including the medial and lateral parietal, medial prefrontal, and medial and lateral temporal regions [58]. These areas show increased activity when the body is at rest, not engaged in movement or cognitive tasks [58]. Previous studies have indicated that DMN can reveal diseased states when differences are observed between the healthy and cognitively impaired [16]. However, similar to challenges faced in fMRI studies, establishing a baseline with a single task, such as asking participants to close their eyes, can be challenging [59]. During the eyes-closed condition, participants may have their minds wander, engage in organizational tasks, be concerned about future events, or question the purpose of the EEG assessment. As a result, the condition might not be the most precise method for assessing neurocognitive decline. A more focused task could provide a better comparison between groups, especially when the goal is to determine cortical activation rather than just baseline activity. The use of EEG to assess cognitive status requires further investigation to understand better the neural biomarkers associated with both healthy aging and disease. Although resting-state EEG has produced varied results, many studies concentrate on cognitive tasks and other active participation tests. These task-oriented approaches may be more effective in differentiating between healthy aging and disease states. Using a greater number of electrodes can help reduce errors when employing EEG as an assessment tool. The 14-channel Emotiv EPOC-X headset, while easier to set up and manage with larger populations, has fewer electrodes compared to other systems with more channels [15,60,61,62]. As described in the methodology, the reduction in channels left some participants with only 12 electrodes, limiting spatial data and potentially skewing results toward the remaining electrodes. Additionally, participants completed other tasks outside the scope of this manuscript within one continuous EEG data file, necessitating the trimming and processing of data for each task. Since the software does not test electrode connections between tasks, some electrodes may have lost signal, potentially affecting the results.

This study is novel in its application of the Emotiv EPOC-X 14-channel system for assessing the MCI population. Since its introduction, the Emotiv EPOC-X has been used globally in various applications, including controlling robotic limbs and wheelchairs, user authentication in security systems, and identifying emotional states. Its ease of setup makes portable EEG devices ideal for clinical settings, allowing for comprehensive data collection with minimal setup time [63]. Our study population had a skewed sex distribution, with a higher proportion of females, which aligns with the existing literature showing that females have a higher incidence of dementia in the U.S. and Europe [64]. Therefore, this study’s results are generalizable to a broader population and reflect the typical sex distribution seen in dementia research. A key limitation of our study is the small sample size, as most EEG research on neural biomarkers typically involves larger samples. Many previous studies have focused on changes observed during cognitive tasks, while our study examines a 2-min resting state. The lack of directionality in the resting state limits the extent of observable changes, although longer resting periods may reveal more significant differences than those detected in our study [15,16,32,65,66]. While nonlinear methods, such as differential equations (DEs), could offer additional insights into subtle differences, particularly between MCI and HC, this manuscript does not utilize this approach. We plan to explore DEs in future studies, particularly when applying more directed tasks to better differentiate between cognitive statuses. Moreover, many studies using EEG for similar purposes do not employ the MoCA assessment for group selection but instead utilize other assessment methods (e.g., Mini-Mental State Examination, Dementia Rating Scale, Neuroimaging/Machine Learning, and CSF/Blood biomarkers) [67,68,69,70,71]. Aside from the study’s limitations, a key strength and innovative approach was the use of a subjective measure of cognitive decline (MoCA), which is correlated to an objective measure of cognitive function [15,16,60,61,62,69,71].

## 5. Conclusions

While our analysis of the Default Mode Network (DMN) in the eyes-closed resting state shows promise for distinguishing between dementia and healthy controls, future research would benefit from incorporating task-based EEG conditions. These conditions could provide a more controlled environment to detect the subtle changes characteristic of MCI. Given the variability of spontaneous thought patterns during the resting state, task-based EEG may offer clearer insights into early cognitive changes [59]. This uncontrolled variability is particularly problematic when attempting to identify early-stage changes, such as those characteristic of MCI, where differences in network connectivity may be more subtle. Since the primary differences between healthy controls and those with cognitive impairments often emerge in task performance—whether cognitive or motor—eyes-closed DMN analysis alone may fail to capture these functional disruptions [33,42]. Combining DMN analysis with targeted cognitive or motor tasks could provide a more controlled and sensitive approach, improving the ability to categorize stages of cognitive decline.

## Figures and Tables

**Table 1:** EEG participants’ demographics and cognitive assessment scores.

Variable	Healthy Controls (79)	MCI (36)	Dementia (7)	*p*-Value (One-Way ANOVA)
**Demographics**				
Male (*n*)	26	17	2	-
Female (*n*)	53	19	5	-
Education (years)	16.4 ± 3.5	16.1 ± 3.8	12.3 ± 2.1	0.01 *
Age (years)	73.6 ± 5.4	75.6 ± 7.9	77.9 ± 5.8	0.09
Body Mass Index	27.1 ± 6.9	26.5 ± 5.0	28.8 ± 7.6	0.6
**Cognitive Assessments**				
MoCA Score	27.2 ± 1.2 ^a,b^	22.9 ± 1.8 ^a,c^	11.9 ± 4.2 ^b,c^	<0.0001 *
Mini-Cog© Score	4.5 ± 0.9 ^a,b^	3.7 ± 1.2 ^a,c^	0.9 ± 0.9 ^b,c^	<0.0001 *
MIS	7.1 ± 1.2 ^a,b^	5.4 ± 2.3 ^a,c^	2.3 ± 3.1 ^b,c^	<0.0001 *
CDQE	48.1 ± 6.4 ^b^	46.3 ± 11.5 ^c^	60.4 ± 11.3 ^b,c^	0.0005 *
AD8	0.7 ± 1.2 ^b^	1.3 ± 1.7 ^c^	4.9 ± 2.7 ^b,c^	<0.0001 *

* Significant differences; ^a^ Significant differences between HC and MCI; ^b^ Significant differences between HC and D; ^c^ Significant differences between D and MCI.

## Data Availability

The data supporting this study’s findings are available on request from the corresponding authors, N.D.S./S.J. However, the data are not publicly available because they contain information that could compromise the privacy of research participants.

## Abbreviations

The following abbreviations are used in this manuscript:

EEG	Electroencephalography
MCI	Mild Cognitive Impairment
PSD	Power Spectral Density
MoCA	Montreal Cognitive Assessment
MIS	Memory Impairment Screen
CDQE	Questionnaire on Cognitive Decline in the Elderly
IQCODE	Short Form of the Informant Questionnaire on Cognitive Decline
AD8	Eight-Item Informant Interview to Differentiate Aging and Dementia
HC	Healthy Controls
D	Dementia
CMS	Common Mode Sense
DRL	Driven Right Leg
ICA	Independent Component Analysis
DMN	Default Mode Network

## References

[1] Wiley Periodicals LLC on behalf of Alzheimer’s Association (2024). 2024 Alzheimer’s Disease Facts and Figures. Alzheimer’s Dement..

[2] Brookmeyer R., Abdalla N., Kawas C.H., Corrada M.M. (2018). Forecasting the Prevalence of Preclinical and Clinical Alzheimer’s Disease in the United States. Alzheimer’s Dement..

[3] Deardorff W.J., Feen E., Grossberg G.T. (2015). The Use of Cholinesterase Inhibitors Across All Stages of Alzheimer’s Disease. Drugs Aging.

[4] Olivares D., Deshpande V.K., Shi Y., Lahiri D.K., Greig N.K., T Rogers J., Huang X. (2012). N-Methyl D-Aspartate (NMDA) Receptor Antagonists and Memantine Treatment for Alzheimer’s Disease, Vascular Dementia and Parkinson’s Disease. Curr. Alzheimer Res..

[5] Scott K.R., Barrett A.M. (2007). Dementia Syndromes: Evaluation and Treatment. Expert Rev. Neurother..

[6] van der Flier W.M., Tijms B.M. (2023). Treatments for AD: Towards the Right Target at the Right Time. Nat. Rev. Neurol..

[7] McKhann G.M., Knopman D.S., Chertkow H., Hyman B.T., Jack C.R., Kawas C.H., Klunk W.E., Koroshetz W.J., Manly J.J., Mayeux R. (2011). The Diagnosis of Dementia Due to Alzheimer’s Disease: Recommendations from the National Institute on Aging-Alzheimer’s Association Workgroups on Diagnostic Guidelines for Alzheimer’s Disease. Alzheimer’s Dement..

[8] Lee J. (2023). Mild Cognitive Impairment in Relation to Alzheimer’s Disease: An Investigation of Principles, Classifications, Ethics, and Problems. Neuroethics.

[9] Sperling R.A., Aisen P.S., Beckett L.A., Bennett D.A., Craft S., Fagan A.M., Iwatsubo T., Jack C.R., Kaye J., Montine T.J. (2011). Toward Defining the Preclinical Stages of Alzheimer’s Disease: Recommendations from the National Institute on Aging-Alzheimer’s Association Workgroups on Diagnostic Guidelines for Alzheimer’s Disease. Alzheimer’s Dement..

[10] Farias S.T., Mungas D., Reed B.R., Harvey D., DeCarli C. (2009). Progression of Mild Cognitive Impairment to Dementia in Clinic- vs Community-Based Cohorts. Arch. Neurol..

[11] Jack C.R., Bennett D.A., Blennow K., Carrillo M.C., Dunn B., Haeberlein S.B., Holtzman D.M., Jagust W., Jessen F., Karlawish J. (2018). NIA-AA Research Framework: Toward a Biological Definition of Alzheimer’s Disease. Alzheimer’s Dement..

[12] Scheltens P., De Strooper B., Kivipelto M., Holstege H., Chételat G., Teunissen C.E., Cummings J., van der Flier W.M. (2021). Alzheimer’s Disease. Lancet.

[13] van Maurik I.S., Vos S.J., Bos I., Bouwman F.H., Teunissen C.E., Scheltens P., Barkhof F., Frolich L., Kornhuber J., Wiltfang J. (2019). Biomarker-Based Prognosis for People with Mild Cognitive Impairment (ABIDE): A Modelling Study. Lancet Neurol..

[14] Farina F.R., Emek-Savaş D.D., Rueda-Delgado L., Boyle R., Kiiski H., Yener G., Whelan R. (2020). A Comparison of Resting State EEG and Structural MRI for Classifying Alzheimer’s Disease and Mild Cognitive Impairment. Neuroimage.

[15] Meghdadi A.H., Stevanović Karić M., McConnell M., Rupp G., Richard C., Hamilton J., Salat D., Berka C. (2021). Resting State EEG Biomarkers of Cognitive Decline Associated with Alzheimer’s Disease and Mild Cognitive Impairment. PLoS ONE.

[16] Smailovic U., Johansson C., Koenig T., Kåreholt I., Graff C., Jelic V. (2021). Decreased Global EEG Synchronization in Amyloid Positive Mild Cognitive Impairment and Alzheimer’s Disease Patients—Relationship to APOE Ε4. Brain Sci..

[17] Vecchio F., Miraglia F., Iberite F., Lacidogna G., Guglielmi V., Marra C., Pasqualetti P., Tiziano F.D., Rossini P.M. (2018). Sustainable Method for Alzheimer Dementia Prediction in Mild Cognitive Impairment: Electroencephalographic Connectivity and Graph Theory Combined with Apolipoprotein E. Ann. Neurol..

[18] Vecchio F., Miraglia F., Alù F., Menna M., Judica E., Cotelli M., Rossini P.M. (2020). Classification of Alzheimer’s Disease with Respect to Physiological Aging with Innovative EEG Biomarkers in a Machine Learning Implementation. J. Alzheimer’s Dis..

[19] Cassani R., Falk T.H., Fraga F.J., Cecchi M., Moore D.K., Anghinah R. (2017). Towards Automated Electroencephalography-Based Alzheimer’s Disease Diagnosis Using Portable Low-Density Devices. Biomed. Signal Process. Control.

[20] Hatz F., Hardmeier M., Benz N., Ehrensperger M., Gschwandtner U., Rüegg S., Schindler C., Monsch A.U., Fuhr P. (2015). Microstate Connectivity Alterations in Patients with Early Alzheimer’s Disease. Alzheimer’s Res. Ther..

[21] Monllor P., Cervera-Ferri A., Lloret M.-A., Esteve D., Lopez B., Leon J.-L., Lloret A. (2021). Electroencephalography as a Non-Invasive Biomarker of Alzheimer’s Disease: A Forgotten Candidate to Substitute CSF Molecules?. Int. J. Mol. Sci..

[22] Musaeus C.S., Engedal K., Høgh P., Jelic V., Mørup M., Naik M., Oeksengaard A.-R., Snaedal J., Wahlund L.-O., Waldemar G. (2018). EEG Theta Power Is an Early Marker of Cognitive Decline in Dementia Due to Alzheimer’s Disease. J. Alzheimer’s Dis..

[23] Babiloni C., Arakaki X., Bonanni L., Bujan A., Carrillo M.C., Del Percio C., Edelmayer R.M., Egan G., Elahh F.M., Evans A. (2021). EEG Measures for Clinical Research in Major Vascular Cognitive Impairment: Recommendations by an Expert Panel. Neurobiol. Aging.

[24] Besthorn C., Förstl H., Geiger-Kabisch C., Sattel H., Gasser T., Schreiter-Gasser U. (1994). EEG Coherence in Alzheimer Disease. Electroencephalogr. Clin. Neurophysiol..

[25] Moretti D.V., Pievani M., Fracassi C., Binetti G., Rosini S., Geroldi C., Zanetti O., Rossini P.M., Frisoni G.B. (2009). Increase of Theta/Gamma and Alpha3/Alpha2 Ratio Is Associated with Amygdalo-Hippocampal Complex Atrophy. J. Alzheimer’s Dis..

[26] Moretti D.V., Prestia A., Fracassi C., Binetti G., Zanetti O., Frisoni G.B. (2012). Specific EEG Changes Associated with Atrophy of Hippocampus in Subjects with Mild Cognitive Impairment and Alzheimer’s Disease. Int. J. Alzheimer’s Dis..

[27] Nardone R., Sebastianelli L., Versace V., Saltuari L., Lochner P., Frey V., Golaszewski S., Brigo F., Trinka E., Höller Y. (2018). Usefulness of EEG Techniques in Distinguishing Frontotemporal Dementia from Alzheimer’s Disease and Other Dementias. Dis. Markers.

[28] Schumacher J., Taylor J.-P., Hamilton C.A., Firbank M., Cromarty R.A., Donaghy P.C., Roberts G., Allan L., Lloyd J., Durcan R. (2020). Quantitative EEG as a Biomarker in Mild Cognitive Impairment with Lewy Bodies. Alzheimer’s Res. Ther..

[29] Stam C.J., Van Der Made Y., Pijnenburg Y.A.L., Scheltens P. (2003). EEG Synchronization in Mild Cognitive Impairment and Alzheimer’s Disease. Acta Neurol. Scand..

[30] McBride J.C., Zhao X., Munro N.B., Smith C.D., Jicha G.A., Hively L., Broster L.S., Schmitt F.A., Kryscio R.J., Jiang Y. (2014). Spectral and Complexity Analysis of Scalp EEG Characteristics for Mild Cognitive Impairment and Early Alzheimer’s Disease. Comput. Methods Programs Biomed..

[31] Vecchio F., Babiloni C., Lizio R., De Vico Fallani F., Blinowska K., Verrienti G., Frisoni G., Rossini P.M. (2013). Resting State Cortical EEG Rhythms in Alzheimer’s Disease. Supplements to Clinical Neurophysiology.

[32] Nasreddine Z.S., Phillips N.A., Bédirian V., Charbonneau S., Whitehead V., Collin I., Cummings J.L., Chertkow H. (2005). The Montreal Cognitive Assessment, MoCA: A Brief Screening Tool for Mild Cognitive Impairment. J. Am. Geriatr. Soc..

[33] Mini-Cog© Quick Screening for Early Dementia Detection. https://mini-cog.com/.

[34] Buschke H., Kuslansky G., Katz M., Stewart W.F., Sliwinski M.J., Eckholdt H.M., Lipton R.B. (1999). Screening for Dementia with the Memory Impairment Screen. Neurology.

[35] Jorm A.F. (1994). A Short Form of the Informant Questionnaire on Cognitive Decline in the Elderly (IQCODE): Development and Cross-Validation. Psychol. Med..

[36] Jorm A.F. (1996). Assessment of Cognitive Impairment and Dementia Using Informant Reports. Clin. Psychol. Rev..

[37] Jorm A.F., Christensen H., Henderson A.S., Jacomb P.A., Korten A.E., Mackinnon A. (1996). Informant Ratings of Cognitive Decline of Elderly People: Relationship to Longitudinal Change on Cognitive Tests. Age Ageing.

[38] Jorm A.F., Broe G.A., Creasey H., Sulway M.R., Dent O., Fairley M.J., Kos S.C., Tennant C. (1996). Further Data on the Validity of the Informant Questionnaire on Cognitive Decline in the Elderly (IQCODE). Int. J. Geriatr. Psychiatry.

[39] Jorm A.F. (2004). The Informant Questionnaire on Cognitive Decline in the Elderly (IQCODE): A Review. Int. Psychogeriatr..

[40] Rosenzweig A. (2024). Montreal Cognitive Assessment (MoCA) Test for Dementia.

[41] Cordell C.B., Borson S., Boustani M., Chodosh J., Reuben D., Verghese J., Thies W., Fried L.B. (2013). Alzheimer’s Association Recommendations for Operationalizing the Detection of Cognitive Impairment during the Medicare Annual Wellness Visit in a Primary Care Setting. Alzheimer’s Dement..

[42] Burton J.K., Fearon P., Noel-Storr A.H., McShane R., Stott D.J., Quinn T.J. (2021). Informant Questionnaire on Cognitive Decline in the Elderly (IQCODE) for the Detection of Dementia within a Secondary Care Setting. Cochrane Database Syst. Rev..

[43] Galvin J.E., Roe C.M., Xiong C., Morris J.C. (2006). Validity and Reliability of the AD8 Informant Interview in Dementia. Neurology.

[44] Svensson A., Granvik E., Sjögren Forss K. (2020). Performance of the Eight-Item Informant Interview to Differentiate Aging and Dementia within a Context Similar to the Swedish Primary Healthcare Sector: A Systematic Review of Diagnostic Test Accuracy Studies. Scand. J. Prim. Health Care.

[45] Kounios J., Fleck J.I., Zhang F., Oh Y. (2024). Brain-Age Estimation with a Low-Cost EEG-Headset: Effectiveness and Implications for Large-Scale Screening and Brain Optimization. Front. Neuroergon..

[46] Delorme A., Makeig S. (2004). EEGLAB: An Open Source Toolbox for Analysis of Single-Trial EEG Dynamics Including Independent Component Analysis. J. Neurosci. Methods.

[47] Chang C.-Y., Hsu S.-H., Pion-Tonachini L., Jung T.-P. (2018). Evaluation of Artifact Subspace Reconstruction for Automatic EEG Artifact Removal. Proceedings of the 2018 40th Annual International Conference of the IEEE Engineering in Medicine and Biology Society (EMBC).

[48] Pion-Tonachini L., Kreutz-Delgado K., Makeig S. (2019). ICLabel: An Automated Electroencephalographic Independent Component Classifier, Dataset, and Website. Neuroimage.

[49] Del Percio C., Lopez S., Noce G., Lizio R., Tucci F., Soricelli A., Ferri R., Nobili F., Arnaldi D., Famà F. (2023). What a Single Electroencephalographic (EEG) Channel Can Tell Us about Alzheimer’s Disease Patients with Mild Cognitive Impairment. Alzheimer’s Dement..

[50] Krothapalli M., Buddendorff L., Yadav H., Schilaty N.D., Jain S. (2024). From Gut Microbiota to Brain Waves: The Potential of the Microbiome and EEG as Biomarkers for Cognitive Impairment. Int. J. Mol. Sci..

[51] Chen G., Zhao M., Yang K., Lin H., Han C., Wang X., Han Y. (2021). Education Exerts Different Effects on Cognition in Individuals with Subjective Cognitive Decline and Cognitive Impairment: A Population-Based Study. J. Alzheimer’s Dis..

[52] Zahodne L.B., Stern Y., Manly J.J. (2015). Differing Effects of Education on Cognitive Decline in Diverse Elders with Low versus High Educational Attainment. Neuropsychology.

[53] Flores-Sandoval A.A., Davila-Pérez P., Buss S.S., Donohoe K., O’Connor M., Shafi M.M., Pascual-Leone A., Benwell C.S.Y., Fried P.J. (2023). Spectral Power Ratio as a Measure of EEG Changes in Mild Cognitive Impairment Due to Alzheimer’s Disease: A Case-Control Study. Neurobiol. Aging.

[54] Newson J.J., Thiagarajan T.C. (2019). EEG Frequency Bands in Psychiatric Disorders: A Review of Resting State Studies. Front. Hum. Neurosci..

[55] Engels M.M.A., Hillebrand A., Van Der Flier W.M., Stam C.J., Scheltens P., Van Straaten E.C.W. (2016). Slowing of Hippocampal Activity Correlates with Cognitive Decline in Early Onset Alzheimer’s Disease. An MEG Study with Virtual Electrodes. Front. Hum. Neurosci..

[56] Jeong J. (2004). EEG Dynamics in Patients with Alzheimer’s Disease. Clin. Neurophysiol..

[57] Raichle M.E. (2015). The Brain’s Default Mode Network. Annu. Rev. Neurosci..

[58] Stark C.E.L., Squire L.R. (2001). When Zero Is Not Zero: The Problem of Ambiguous Baseline Conditions in FMRI. Proc. Natl. Acad. Sci. USA.

[59] Aljalal M., Aldosari S.A., Molinas M., Alturki F.A. (2024). Selecting EEG Channels and Features Using Multi-Objective Optimization for Accurate MCI Detection: Validation Using Leave-One-Subject-out Strategy. Sci. Rep..

[60] D’Atri A., Scarpelli S., Gorgoni M., Truglia I., Lauri G., Cordone S., Ferrara M., Marra C., Rossini P.M., De Gennaro L. (2021). EEG Alterations during Wake and Sleep in Mild Cognitive Impairment and Alzheimer’s Disease. iScience.

[61] Martin T., Giordani B., Kavcic V. (2022). EEG Asymmetry and Cognitive Testing in MCI Identification. Int. J. Psychophysiol..

[62] Sabio J., Williams N.S., McArthur G.M., Badcock N.A. (2024). A Scoping Review on the Use of Consumer-Grade EEG Devices for Research. PLoS ONE.

[63] Beam C.R., Kaneshiro C., Jang J.Y., Reynolds C.A., Pedersen N.L., Gatz M. (2018). Differences between Women and Men in Incidence Rates of Dementia and Alzheimer’s Disease. J. Alzheimer’s Dis..

[64] Barry R.J., Clarke A.R., Johnstone S.J., Magee C.A., Rushby J.A. (2007). EEG Differences between Eyes-Closed and Eyes-Open Resting Conditions. Clin. Neurophysiol..

[65] Barry R.J., De Blasio F.M. (2017). EEG Differences between Eyes-Closed and Eyes-Open Resting Remain in Healthy Ageing. Biol. Psychol..

[66] Economou A., Pavlou D., Beratis I., Andronas N., Papadimitriou E., Papageorgiou S.G., Yannis G. (2020). Predictors of Accidents in People with Mild Cognitive Impairment, Mild Dementia Due to Alzheimer’s Disease and Healthy Controls in Simulated Driving. Int. J. Geriatr. Psychiatry.

[67] Ilardi C.R., Menichelli A., Michelutti M., Cattaruzza T., Manganotti P. (2023). Optimal MoCA Cutoffs for Detecting Biologically-Defined Patients with MCI and Early Dementia. Neurol. Sci..

[68] Jiao B., Li R., Zhou H., Qing K., Liu H., Pan H., Lei Y., Fu W., Wang X., Xiao X. (2023). Neural Biomarker Diagnosis and Prediction to Mild Cognitive Impairment and Alzheimer’s Disease Using EEG Technology. Alzheimer’s Res. Ther..

[69] Khazaee A., Ebrahimzadeh A., Babajani-Feremi A. (2017). Classification of Patients with MCI and AD from Healthy Controls Using Directed Graph Measures of Resting-State FMRI. Behav. Brain Res..

[70] Petersen R.C., Smith G.E., Waring S.C., Ivnik R.J., Tangalos E.G., Kokmen E. (1999). Mild Cognitive Impairment. Arch. Neurol..

[71] Schilaty N.D., Nagelli C., Hewett T.E. (2016). Use of Objective Neurocognitive Measures to Assess the Psychological States That Influence Return to Sport Following Injury. Sports Med..

